# An Inquiry, Critical and Experimental, into the Pathology of Fever

**Published:** 1853-07

**Authors:** N. S. Davis

**Affiliations:** Professor of Pathology, Practice of Medicine, and Clinical Medicine, in Rush Medical College; and one of the Physicians to the Ill. Gen. Hospital


*An Inquiry. Critical and Experimental, into the Pathology of Fever* By N. S. Davis M. D. Professor of Pathology, Practice of Medicine, and Clinical Medicine, in Rush Medical College; and one of the Physicians to the Ill. Gen. Hospital.

[Continued from Page 112.]

*Present Appearance or Symptoms.*—The countenance is dull and inexpressive; face bloated; skin sallow; lips and tongue pale; eyes clear; tongue and mouth clean and moist; universal œdema of the cellular tissue to such an extent that pressure leaves pits on any part of the body and extremities, the latter being much swollen; abdominal dulness and fluctuation, indicating moderate effusion into the cavity of the peritoneum; hepatic dulness appeared to occupy a less space than natural, leading to suspicion of incipient cirrhosis; certainly no evidence of enlargement of the liver or spleen; thoracic resonance good, and respiratory murmur normal, except feebler than usual; rythm and sounds of the heart normal, except a plain anemic bellow's murmur; pulse small, soft, and 80 per minute; bowels regular and fœces natural in appearance; urine scanty and loaded with urate of ammonia, giving it a clouded appearance; appetite impaired; muscular weakness very great; position recumbent and dorsal.

*History.*—The patient has had intermittent fever during the last two months, at least, but cannot learn the exact length of time. He has still regular paroxysms every morning, exhibiting a well marked chill, succeeded by a hot stage, during which the pulse is much excited and the anæmic bellow's murmur of the heart very loud and distinct.

*Treatment.*—Viewing the case as one of extreme loss of tonicity in the solids, and great impoverishment of the blood, from a protracted continuance of that peculiar disturbance of the vital properties, which constitutes the essential pathology of periodical fever, without any satisfactory evidence of local visceral disease, the plain indications of treatment, were, first, to interrupt the paroxysms, and second, to improve the tonicity of the solids and the composition of the fluids.

To fulfil the first, he was ordered chloride of sodium ʒj., and sulphate of quinine 12 grs., to be divided into four powders, and one given at such intervals that the whole would be taken before the time for the next chill.

Reel 178 -10 - 5 CONT

Nov. 28th. Had a chill this morning, but much lighter than before. Continue same medicine until four more doses are taken, with animal broth well salted for nourishment.

Nov. 29th. No chill or fever this morning. All other symptoms as at first. Ordered a powder composed of chloride of sodium 10 grs., sulph. quinine 2 grs., carb. of iron 3 grs., to be given four times a day, and continue same diet.

Nov. 30th. No return of chills or fever; otherwise all the symptoms, including the debility, the dropsical effusions &c., were the same as on the day of admission. I now took from his arm, in a clean glass cup, 1448 grs. Troy weight, of blood for examination and analysis. He was then ordered a powder, consisting of extract of sanguinis or dried beef's blood, 20 grs. and sulp. quinine, 2 grs., to be taken four times a day, with an infusion of juniper berries and *uva ursi* for a drink. A diet of animal broth, milk and bread was continued. This treatment was steadily persisted in with a pretty uniform improvement in all the symptoms for three weeks, when he was discharged from the hospital without any symptoms of intermittent fever or dropsy, and feeling quite well. I met him again in the street on the 30th of December, when he looked and felt entirely well.

*Examination of the Blood.*—The blood coagulated as quickly as in health; the clot becoming firm, small, floating in a clear serum, and retaining accurately the shape of the vessel containing it, without being either cupped or buffed.

Viewed under the microscope, with an object glass magnifying 850 diameters, the red corpuscles differed in no respect from their appearance in healthy blood, except they were in a slight degree more distended or globular. Occasionally one was seen presenting a shrivelled, irregular, and corrugated appearance, but not more so than is sometimes seen in persons apparently in good health. The white corpuscles were certainly deficient in number, only *one* being seen during an examination of six or eight different fields, and the addition, to some of them, of distilled water and acetic acid for the purpose of rendering them more distinct.

The 1448 grs. of blood, after standing, yielded 1048 grs. of clear serum, and 400 grs. of clot. The latter being carefully washed and the product dried over sulph. acid, under the receiver of an air pump, gave 2 grs. of fibrin.

The 1048 grs. of clear serum, by evaporation and careful drying over the acid in a vacuum, yielded 75 grs. of solid matter, and 973 grs. of water.

The 398 grs. of clot, treated in the same manner, gave 98 grs. of solid matter, and 300 grs. of water.

The 75 grs. of solid matter from the serum, and the 98 grs. from the clot, after digesting twenty-four hours in strong sulphuric ether, yielded to the latter only 1 gr. of soluble matter. The solid matter of the serum left, after incineration in a porcelain crucible, 1,5 grs. of ash. That of the clot yielded, by the same process, 4,5 grs. of ash.

The combined results may be stated in tabular form, as follows, viz :

WHOLE AMOUNT OF BLOOD EXAMINED, 1448 GRAINS.

| | |
|---|---|
| Water, | 1273, grains. |
| Red Corpuscles, | 72,1 " |
| Albumen, | 93,9 " |
| Fibrin, | 2, " |
| Matter soluble in Ether, | 1, " |
| Ash or saline matter | 6, " |
| Total | 1448,0 grains. |

PROPORTION IN 1000 PARTS OF BLOOD.

| | |
|---|---|
| Water, | 879,14 parts. |
| Red Corpuscles, | 49,79 " |
| Albumen, | 64,84 " |
| Fibrin, | 1,38 " |
| Matter soluble in Ether, | ,69 " |
| Saline matter | 4,14 " |
| Total | 999,98 parts. |

### CASE 2.

The description of this case may be condensed from my note book, as follows, viz : Mr. Sampson, a Swede, aged 23 years, was admitted into the hospital Nov. 28, 1852. Says he has had a quotidian intermittent almost constantly during the last six or eight weeks. At present he is moderately emaciated ; countenance sallow ; lips and tongue pale ; pulse 85 per minute, and easily compressed ; cutaneous and urinary secretions diminished, with slight tendency to diarrhœa, and great muscular weakness. No signs of œdema or of visceral disease, either in the chest or abdomen.

His intermittent paroxysms were promptly arrested by the exhibition of chloride of sodium and sulph. of quinine, in the same manner as in case No. 1. On the fourth day after admission, no chills having recurred since the second day, I took 1410 grs. of blood from his arm for examination, and ordered him extract of sanguinis, 15 grs., four times a day, with a nutritious diet. He continued this treatment steadily, with uniform improvement, and was discharged apparently well near the end of the third week after admission.

*Examination of the Blood.*—In coagulating and under the microscope, this blood presented the same phenomena and appearances as No. 1. The clot was larger in proportion to the serum, but there was the same shape, firmness, &c., and the same deficiency of white corpuscles. The different steps of analysis were conducted in all respects the same as in the former case, and the result stated in tabular form is as follows, viz :

WHOLE AMOUNT OF BLOOD EXAMINED, 1410 GRS.

| | | |
|---|---|---|
| Water, | 1182,50 | grains. |
| Red corpuscles, | 116,74 | " |
| Albumen, | 98,26 | " |
| Fibrin, | 3.50 | " |
| Matter soluble in ether, | 1.00 | " |
| Ash or saline matter, | 8 00 | " |
| Total, | 1410,00 | " |

PROPORTION PER 1000 PARTS.

| | | |
|---|---|---|
| Water, | 838,65 | parts. |
| Red corpuscles, | 82,79 | " |
| Albumen, | 69 68 | " |
| Fibrin, | 2,48 | " |
| Fatty matter, | 71 | " |
| Saline matter, | 5.67 | " |
| Total, | 999 98 | " |

### CASE 3.

December 9, 1852, Mr. Gibbs, a German, aged 30 years, was admitted into the General Hospital in the advanced stage of enteric or typhoid fever. It is now three weeks since the attack commenced, or since he was confined to his bed. He is moderately emaciated; his countenance dull and inexpressive; his face and lips deep red; eyes heavy; mouth dry, with some brown sordes around the teeth; tongue red, pointed, smooth and dry; dulness of hearing; moderate delirium, especially during the night; skin dry, harsh, and above the natural temperature; pulse 112 per minute, soft and easily compressed; abdomen covered with patches of a red papular eruption, full and tympanitic; urine scanty; discharges from the bowels average three or four per day, are thin, brown, and offensive, and during the last eighteen hours they have been more frequent, copious, and tinged with blood. I opened a vein and took from his arm 1634 grs. of blood for examination.

*Treatment.*—The patient was ordered a blister over the abdomen, and an emulsion, consisting of oil of turpentine ʒij, tinct. opii ʒij, gum Arabic and white sugar, each ʒiij, rubbed thoroughly together, with the addition of water ℥ij; of which a fluid drachm was to be given every three hours, with beef tea well salted for nourishment, and sponging of the surface with warm water every afternoon.

This treatment was continued, without alteration, four days, with a gradual improvement of all the symptoms; three grains of quinine were then added to each morning dose of the emulsion, and in four days more convalescence was fully established. Soon after convalescence, an abscess of moderate size appeared on one arm, was opened and discharged considerable unhealthy pus, but soon healed. December 30th, he was discharged from the Hospital quite well.

*Examination of the Blood.*—The blood, as it flowed from the vein, was of a very dark color; and, examined under a magnifying power of 850 diameters, soon after it was drawn, the red corpuscles were seen much more variable in size, and more irregular in appearance than in healthy blood; one-third of the whole number brought into view were much below the ordinary size, while a few appeared nearly as large as the white corpuscles; and the same disparity in size was maintained after the addition of distilled water. A much larger number were also irregular, shrivelled or plaited, than in healthy blood. No white corpuscles were seen on any of the fields examined, though they were diligently sought for. The blood coagulated in the vessel very slowly and in partially disconnected masses, which, however, slowly united into a uniform clot, preserving the shape of the cup, without any buff, and less firm and compact than the clots of No 1 and 2.

After standing 36 hours, the 1634 grs. of blood gave 675 grs. of clear serum, and 959 grs. of clot. The several proximate constituents having been carefully separated, in the same manner as in the preceding analyses, presented the following results, viz:—

THE WHOLE AMOUNT OF BLOOD ANALIZED, 1634 GRS.

| | | |
|---|---|---|
| Water, - - - - - - | 1327,50 | grains. |
| Red corpuscles, - - - - - | 174,47 | " |
| Albumen, - - - - - | 109.93 | " |
| Fibrin, - - - - - - - | 4,50 | " |
| Matter soluble in ether. - - - | 1.10 | " |
| Ash or saline matter, - - - - - | 16 50 | " |
| Total, - - - - - | 1634.00 | " |

PROPORTION IN 1000 PARTS.

| | | |
|---|---|---|
| Water, - - - - - - - | 812.42 | parts. |
| Red corpuscles, - - - - | 106,77 | " |
| Albumen, - - - - - - | 67 27 | " |
| Fibrin, - - - - - - | 2 75 | " |
| Fatty matter, - - - - - - | 67 | " |
| Saline, - - - - - - - - | 10 09 | " |
| Total, - - - - - - | 999 97 | " |

*Remarks.*—Cases No. 1 and 2 were selected as fair specimens of the uncontrolled influence of intermittent fever on the solids and fluids of the human system, when protracted and uncomplicated with any appreciable local lesions. They represent different stages in the progress of the same morbid actions, and the results of the analyses of the blood, show simply different *degrees* of impoverishment of the same proximate elements.

The first presents one of the most extreme cases of simple impairment of the vital properties of the solids and impoverishment of the fluids, that I have ever seen recover. The second, represents a much more numerous and common class of cases.

The third case was a very characteristic example of the most common form of continued fever. And certainly the blood, viewed microscopically and analytically, presents characteristics as strikingly diverse from the two first specimens, as do the symptoms of the one form of fever from those of the other. In the two first specimens the principal morbid changes consist in absolute impoverishment of the essential constituents of the blood, with only slight apparent alteration in their qualities; but in the third specimen, the actual impoverishment was slight, while impairment of the qualities or properties of the several constituents was strikingly exhibited.

One interesting feature was presented by all the specimens; which was the paucity of white corpuscles. Whether this is a uniform characteristic of the blood, in cases of idiopathic fevers, uncomplicated with local inflammations, can only be determined by a large number of observations in each form of disease.

In one respect the condition of the blood in the first case just detailed, does not agree fully with the general conclusions arrived at by M. M. Bacquerel and Rodier, in their recent elaborate memoir on diseases of the blood, read to the Academy of Sciences at Paris. They say that, "the *diminution of albumen* produces dropsy; speaking generally, *dropsy* is the symptom of diminution of *albumen*." I have met with very few cases of disease, which presented a more universal tendency to, or development of dropsical effusions and infiltrations, than the one just alluded to; and yet the analysis of his blood showed very little deviation from the normal proportion of albumen; scarcely more, in fact, than in both the other cases, neither of which manifested any symptoms of dropsy whatever. Instead of coinciding with the assertion that "*most dropsies, called essential*, are due *to a diminution of the albumen*," my own observations and analyses compel me to regard the diminution of albumen as a coincident rather than an essential cause. The loss of albumen, like every other change by which the solid constituents are lessened in proportion to the aqueous element, doubtless predisposes to dropsical effusions, but is not of itself the efficient cause. If it were so, more or less dropsical effusion should accompany every case of deficient albumen, which certainly has not been the fact within the sphere of my observation; on the contrary, in reviewing all my examinations of the blood taken from fever patients at different stages of the disease, I have not been able to trace any uniform connection between the proportion of albumen and the development of dropsy. I have observed as a general rule, that œdematous or dropsical symptoms supervened much the most frequently, in those cases characterized in their course by exalted susceptibility or high excitement, with diminished tonicity or vital affinity. These characteristics are much more constantly present in the periodical than in the continued forms of fever, and most of all in the eruptive or exanthematous class. From this, together with the fact that we sometimes meet with great impoverishment of the blood, including diminution of the albumen, without any dropsy, and in other instances dropsy without any such impoverishment; I have been induced to regard the *essential* cause of dropsy as an impaired state of that elementary property of the solids called *tonicity* or vital affinity; and that the diminution of albumen, or any other kind of impoverishment of the blood, acted only as a coincident circumstance, which, by lessening the *consistency* of the blood, aggravated mechanically the tendency to effusion or infiltration. In reference to the changes which the blood undergoes in the progress of fevers, Dr. Drake, in his recent Systematic Treatise, remarks, "I do not know that any experiments have been made on the relative proportion of the proximate elements of the blood, in *our* autumnal fever. Andral and Gavarret, in the hospitals of Paris, made such experiments on the blood of seven patients laboring under intermittents of long standing; and found the mean proportion of fibrin to be *three and a third* of one thousand parts, the normal quantity being three. As many chronic cases of the fever are made such by inflammation of some organ, we may presume that in these cases some were complicated with such inflammations. As to the other proximate elements of the blood, the solid residue of the serum was eighty parts in the thousand, the natural proportion; but the blood corpuscles were, on an average, one hundred and four parts in the thousand, while one hundred and twenty seven is the normal number. Thus, it appears that protracted intermittents produce impoverishment of the blood —spanemia—the condition present in chlorosis; and this accounts in part for the peculiar hue and puffy visage of old ague patients, who so closely simulate chlorosis in their appearance."—See page 820. The two analyses of blood from patients laboring under intermittent fever, which we have given, differ from those of

Andral and Gaverret, in showing a less proportion of fibrin as well as of almost all the other solid constituents of the blood.

For further details on this subject, see Appendix note A.

Notwithstanding the obvious and essential differences between the two great classes of fever now under consideration, they are susceptible of a close approximation, if not an absolute conversion of one into the other.

It is well known that protracted and continuous exercise ultimately exhausts the contractility of the voluntary muscular fibres, and they cease to act until a period of repose has restored their wonted susceptibility. In the same manner, when the over-excited organic actions, which characterize the remittent form of fever, continue unchecked for a considerable period, and the blood become correspondingly impoverished and over-charged with effete matter of a depressing character, the previously exalted susceptibility of the tissues becomes subdued and depressed to the same degree as in the originally Typhoid form of disease. This change is most apt to occur during the third week of the disease, and is marked by a decided diminution of the force of the pulse and capillary circulation; by greater dryness of the respiratory and cutaneous surfaces, inducing more dryness of the tongue and lips with accumulating sordes around the teeth; by greater obtusness of all the nervous functions, and by a more unsteady or tremulous action, of the muscular structures. When these symptoms supervene it is a common remark, that the patient has "fallen into a *Typhoid* state"; or that the fever, previously remittent, has now assumed the Typoid form. This change from the periodical to the continued type of fever, resulting from the ultimate exhaustion or depression of the vital susceptibility, is by no means of unfrequent occurrence; but the reverse change, from the continued type to the true periodical, is so rare that I have never seen a well marked case of the kind. The reason is obvious. The transition from a higher to a lower grade of action, from an exalted to a depressed state of the vital forces, is a natural result of protracted morbid action.

But to pass from depression to exaltation from mere continuance of the diseased processes, would be very much like resting a weary limb by protracting its exercise.

The foregoing views of the essential pathology of fevers, are much strengthened by the phenomena of eruptive fevers, and those properly styled symptomatic. The first arise directly and indisputably from the presence of some virus or poison in the blood, which by its general distribution throughout the whole system, and its irritating qualities, strongly exalts the elementary susceptibilty and thereby rapidly developes an active grade of irritative fever. This continues generally without remission or abatement, until the original cause by some special affinity for the cutaneous tissue, finds in it a lodgement, thereby relieving in a marked degree the general system until it is secondarily disturbed from the severity of the inflammations established in the cutaneous surface. On the other hand, general fibrile symptoms never arise directly from the existence of local inflammation, until the latter, by its extent and persistence, so far interferes with the equality of the circulation of the blood, or with some excretory function which causes the latter to become impregnated with retained effete matter, as to disturb the universal susceptibility of the tissues. Generally, both these modes of disturbing the elementary properties co-operate in establishing general fever, in connection with local inflammation. The primary increased determination of blood to the inflamed part, leaves a defective and disturbed circulation in all other parts of the body. This defective circulation is equivalent to a diminution of the natural stimulant which, by its action on the inherent organic susceptibility, excites and sustains those primary functions, called nutrition, secretion, calorification, &c.; and hence all these become immediately depressed, giving rise to those feelings of indisposition accompanied by alternations of temperature, sometimes amounting to a well formed chill, which characterize the incipient stage of all severe attacks of inflammation.

But this depression of the nutritive, calorific, and secretory functions, necessarily interferes with the metamorphosis of the tissues and retards depuration of the blood, through the action of the skin, kidneys, lungs, &c., thereby causing a sudden and rapidly increasing change in the quality of the blood, by which it is not only rendered irritating to the delicate lining membrane of the whole vascular system, but also morbidly exciting to the susceptibility of all the tissues. With this change in the quality of the blood and its action on the susceptibility of the tissues, comes a corresponding change in the organic actions. The primary feelings of depression, of blunted sensibilities, and variableness in temperature, rapidly give place to those of excitement, acuteness of sensibility, steadily increased heat, and all the phenomena of irritative or inflammatory fever. Of course the specific grade of the febrile symptoms, will depend upon the extent and location of the organ primarily affected, and the previous condition of the vital properties or forces. If from any cause, these properties, and especially that of tonicity, have been in an impaired state previous to the occurrence of the local inflammation, as is generally the case with those who suffer from the privations of poverty in cities, or from intemperance, there will be manifested early a tendency to a typhoid character. The same is true of the inhabitants of highly malarious districts where intermittents and remittents prevail during the summer and autumn. There remains in the inhabitants of such districts, after the warm season has passed away, such a modification of the elementary conditions of vital action, that the occurrence of an inflammation in any important organ, is accompanied by a less sthenic grade of febrile action, than in inflammations of the same extent occurring in inhabitants of healthier districts of country. Here such inflammations are often promptly amenable to modes of treatment, that would entirely fail in other localities. It is thus, that we find the primary and *essential pathology* of all fevers, whether continued or periodical, whether idiopathic or symptomatic, to consist in a disturbance of the natural condition and mutual relations of the three essential and universal elements of vital action, viz: tonicity or a definite texture, susceptibility, and the stimuli capable of acting on them. A general disturbance of the mutual relations of these three elementary conditions, is as constantly and certainly the primary essential change constituting fever, as is the disturbance of the local capillary circulation, the primary element in inflammation. And here is the true "*bond*," spoken of by the philosophic Holland, "by which to associathe together numerous forms of disease." Here, too, is the "*oneness*" of Copeland, and the "*common principle*," mentioned by Dr. Wood, in the paragraph already quoted at the beginning of this Essay. The common principle, the bond of union, the *oneness*

of all fevers, consists in their origin from disturbance of the same elementary properties and functions; while their diversity consists in the different direction given to the disturbance, by the action of exciting causes possessing specifically different qualities. Thus we have the same vital properties morbidly disturbed in the profound depression of a malignant intermittent as in the most excited paroxysm of an inflammatory remittent, or in the slower and more obtuse form of continued fever. But in the first, both tonicity and susceptibility are profoundly depressed or impaired; in the second, tonicity only is impaired, while susceptibility is exalted to the highest degree; and in the third, susceptibility is depressed or perverted while tonicity is but little altered; and these diverse directions taken by the primary morbid actions, develope in the progress of the disease phenomena and results still more various.

The foregoing views may be briefly stated in tabular form as follows, viz:

### TABLE I.

### ELEMENTARY CONSTITUENTS OF FEVER.

| *Elementary Properties.* | | | *Elementary Functions.* |
|---|---|---|---|
| 1 Tonicity.<br>2 Susceptibility. | from which result in | Nerve tissue | Sensibility.<br>Transmissibility.<br>Innervation. |
| | | Contractile tissue | Contractibility.<br>Mobility. |
| | | Glandular tissue | Secretion. |
| | | All tissues | Nutrition.<br>Absorption.<br>Calorification. |

### TABLE II.

### ELEMENTARY CONSTITUENTS VARIOUSLY COMBINED GIVING RISE TO DIFFERENT TYPES OR FORMS OF FEVER.

| | | |
|---|---|---|
| 1 Elementary properties both increased, | Exaltation of all elementary functions, | True inflammatory fever. |

| | | |
|---|---|---|
| 2 Elementary properties both depressed, | Depression of all elementary functions, | Pernicious or malignant periodical fevers and the more profound cases of Typhus. |
| 3 Tonicity normal or diminished with susceptibility increased, | Increased excitement of elementary functions with diminished force, | True irritative fevers, including the ordinary periodical and eruptive classes. |
| 4 Tonicity normal with susceptibility diminished, | Depression with perversion of the elementary functions, | Ordinary typhus and typhoid fever. |

The foregoing is a brief outline of what I deem to be the true philosophy or essential pathology of fevers.

Though hastily written, it is the result of much patient observation at the bed-side, aided by experiments, reading and reflection. It traces, by a rigid analysis, all fevers to a common starting point; not in a morbid impression on any one tissue, like the mucous membranes, as advocated by Broussais, or the *nervous structures*, as claimed by most British and American writers, but on certain inherent and universal properties of all the tissues; which impression does not invariably consist in simple depression or exaltation of such properties as claimed by Dr. Brown; nor yet in constant exaltation, as represented by Dr. Payne, but it varies in its specific character with every variation in the quality of the cause or causes by which it is made. It is thus, that the intricate and complex phenomena of diseases, so widely different in their tendencies, complications, and results, and yet so analogous in some of their essential phenomena, may be unraveled and comprehended as clearly as any of the simpler processes of nature.

It may be thought by some that these views are founded on the exclusive doctrines of *solidism*, and that the importance of the blood, as an elementary seat of disease, is overlooked. It must be remembered, however, that just so far as the blood contains matter in an actual state of organization and possessed of vital properties, so far the foregoing doctrines apply to it as directly as to any of the solid tissues. That the condition of the blood constitutes a very important element in the pathology of fever, and all other general diseases, there can be no doubt. But this importance consists in its relation to the solids as a universal exciter of organic action, and a ready medium, through which noxious agents may be brought, at once, in contact with all the organs and tissues of the system; a relation that links it much more closely to the causes of diseases, than to their essential pathology. As the blood is the common receptacle for all that passes into the system through the digestive and pulmonary organs, and all that is returned from the molecular changes in the tissues through the absorbents, it necessarily constitutes the medium through which all the causes of disease gain access to the human system, except such as act directly and exclusively on the nervous system. It is probable that some of these morbific agents act directly on the constituents of the blood, changing more or less their relative proportion or qualities, while others merely float in the blood as a medium for their mechanical suspension and conveyance to the orga ized tissues. And it is quite probable that some agents act in both ways; altering directly the qualities of the blood and still retaining their own integrity sufficiently to disturb the elementary properties and functions of some one or all of the solid tissues. There are some facts which would lead us to suppose that this is the case in regard to that agent, whatever it may be, which constitutes the efficient cause of intermittents and remittents. The rapid diminution of the red and white corpuscles in the blood of patients laboring under periodical fever, the change in the coloring matter, by which the skin is made to assume that sallow hue, so closely resembling chlorosis, and especially the fact that these phenomena are not unfrequently seen in persons who have lived constantly in malarious districts, but who have never had a single regular paroxysm of fever,—would seem to indicate the existence and action of some cause which acted directly on the blood corpuscles, independent of its further effects on the solid tissues. And it may even admit of some question whether that diminution of *tonicity* in the tissues, which constitutes an important element in the development of every case of intermittent or remittent fever, is not wholly owing to the impaired state of the blood, resulting from the action of the efficient cause on that fluid; while the *increased susceptibility*, which constitutes the other essential element of this variety of fever, results from the direct irritating qualities of the cause, in contact with the organized structures. It will be remembered that the late Dr. Drake and other writers of great experience, assign to the cause of periodical fevers, properties somewhat resembling those of the narcotico-irritant poisons; and yet it will be evident to every careful bed-side observer, that there is neither in the outset nor progress of the disease, any exhibition of a true narcotic or soporific effect.

Instead of sleep or any approach towards narcotism, there is from the very incipient feelings of indisposition to the end of the disease, a sense of debility, a feeling of lassitude both of mind and body, which renders voluntary exertion of any kind, a task, and greatly lessens the power of endurance; while at the same time there is a morbid increase of organic susceptibility, (or irritability, if the reader prefer that term,) which often renders the sleep disturbed, or prevents it altogether, and causes the patient to feel with undue accuteness every outward impression. If as most physiologists of the present day, suppose, the corpuscles of the blood are in some way connected with the absorption of oxygen, and the maintenance of the organic actions in a state of vigorous health and activity, we can perceive very clearly how an agent, introduced into the blood, which is capable of impairing the qualities of such corpuscles and retarding their development, should produce just that lassitude, that impaired tonicity or altered molecular affinity, which so uniformly characterizes the initial steps of periodical fevers. Equally clear it is, that such impairment of the blood and altered molecular affinity would speedily involve a change in all the primary functions, such as innervation, nutrition, secretion, and calorification.

Thus the nervous system would be depressed, nutrition and secretion retarded, and as a necessary accompaniment, the development of caloric would be diminished, thereby leading to a chill, varying from mere alternations of temperature to a prolonged and severe shivering.

If, at the same time, the cause thus acting on the blood and circulating with it through the whole capillary system, retained enough of its original qualities to excite or increase the elementary organic susceptibility, we should have, not the effects of a *narcotico-irritant* agent, but those complex phenomena that result from an impairment of the natural relation between the blood and the elements of the tissues, namely, diminished *tonicity* or true debility, coupled with increased irritability or excitement. And such, it seems to me, are the actual elements that exist and constitute the developing stage of our periodical fevers. Of course, they necessarily involve an early disturbance of all the primary functions, such as innervation, capillary circulation (including nutrition and secretion), and calorification; not in regular and invariable succession, as represented by Dr. Southwood Smith, or as dependencies the two latter on the former—that is, the capillary and calorific disturbance dependent on the nervous depression, as represented by most modern writers—but the whole occurring simultaneously, though not always in an equal ratio or degree, and dependent on a common antecedent pathological condition, already sufficiently described. When the train of morbid action has proceeded far enough to involve the primary functions to such a degree as to produce the phenomena belonging to the cold stage, other disturbing elements are added, which materially aid in arresting the phenomena of depression and in developing those of excitement. Thus the very depression of the primary functions necessarily retards capillary circulation and secretion, inducing an accumulation of blood in the larger vessels and the more vascular organs, while the failure of secretion leaves it impregnated with waste matter that should have been thrown off by the skin, kidneys, &c. This now cóoperates with the irritating quality of the original exciting cause, in speedily exalting the organic susceptibility to such excess as to usher in the hot or fibrile stage If the true irritating quality of the original cause be comparatively feeble, the stage of excitement will be brief, terminating in copious evacuations from the skin and other secreting or exhaling surfaces, thereby exhibiting the phenomena of an ordinary *intermittent.* On the contrary, if the original cause acts with greater intensity, or exhibits more decidedly irritant qualities, the stage of excitement will be more protracted, the secretions partial, being increased in some organs, as when the liver pours out an inordinate quantity of bile, and yet diminished in others, as the skin and kidneys, causing only a partial subsidence of the hot stage, and constituting the common remittent or autumnal bilious fever.

(*Continued next month.*)

